# Клиническая и молекулярно-генетическая характеристика случаев изолированного гипогонадотропного гипогонадизма, обусловленного дефектами рецептора гонадотропин-рилизинг-гормона

**DOI:** 10.14341/probl12746

**Published:** 2021-05-01

**Authors:** Н. А. Макрецкая, М. В. Герасимова, Е. В. Васильев, Н. А. Зубкова, Н. Ю. Калинченко, А. А. Колодкина, В. М. Петров, Т. В. Погода, А. В. Панова, Е. Б. Фролова, А. В. Поляков, А. Н. Тюльпаков

**Affiliations:** Национальный медицинский исследовательский центр эндокринологии; Центр Современной Педиатрии; Национальный медицинский исследовательский центр эндокринологии; Национальный медицинский исследовательский центр эндокринологии; Национальный медицинский исследовательский центр эндокринологии; Национальный медицинский исследовательский центр эндокринологии; Национальный медицинский исследовательский центр эндокринологии; Национальный медицинский исследовательский центр эндокринологии; Национальный медицинский исследовательский центр эндокринологии; Национальный медицинский исследовательский центр; Медико-генетический научный центр им. акад. Н.П. Бочкова; Медико-генетический научный центр им. акад. Н.П. Бочкова

**Keywords:** <i>GNRHR</i>, изолированный гипогонадотропный гипогонадизм, синдром Кальмана, секвенирование нового поколения, аносмия, дигенный дефект, клинический случай

## Abstract

Врожденный гипогонадотропный гипогонадизм (ВГГ) — редкое заболевание, характеризующееся задержкой или отсутствием появления вторичных половых признаков, обусловленное недостаточной выработкой, секрецией или действием гонадотропин-рилизинг-гормона (ГнРГ). Клинически выделяют варианты ВГГ с гипо-/аносмией (синдром Кальмана) и нормосмический гипогонадотропный гипогонадизм. Учитывая многообразие генетических дефектов, отвечающих за развитие ВГГ, применение метода высокоэффективного параллельного секвенирования (NGS) является оптимальным диагностическим подходом, поскольку позволяет одновременно проанализировать несколько генов-кандидатов. Биаллельные мутации в гене GNRHR приводят к развитию гипогонадотропного гипогонадизма с нормосмией. В настоящей работе нами представлено описание 16 пациентов с доказанной резистентностью к ГнРГ, а также оценена частота патогенных вариантов в гене GNRHR в российской популяции.

## Abstract (en)

Congenital hypogonadotropic hypogonadism (CHH) is a rare disorder characterised by lack of pubertal development and infertility, due to deficient production, secretion or action of gonadotropin-releasing hormone (GnRH). Clinically, there are variants of CHH with hypo-/anosmia (Kalman syndrome) and normosmic hypogonadotropic hypogonadism. Given a  growing list of gene mutations accounting for CHH, the application of next generation sequencing (NGS) comprises an excellent molecular diagnostic approach because it enables the simultaneous evaluation of many genes. Biallelic mutations in GNRHR gene lead to the development of hypogonadotropic hypogonadism with normosmia. In this paper, we describe 16 patients with proven GnRH resistance and estimate the frequency of pathogenic variants in the GNRHR gene in the Russian population.

## АКТУАЛЬНОСТЬ

Врожденный гипогонадотропный гипогонадизм (ВГГ) — снижение функции половых желез, в основе которого лежит нарушение синтеза, секреции или действия гонадотропин-рилизинг-гормона (ГнРГ) — ключевого нейрорегулятора репродуктивной системы млекопитающих. ВГГ может проявляться изолированным нарушением функции ГнРГ, сочетаться с недостаточностью других гормонов аденогипофиза (врожденный гипопитуитаризм) или быть частью синдромов. Клинически принято также выделять вариант ВГГ с гипо-/аносмией (синдром Кальмана), патогенез которого связывают с нарушением миграции ГнРГ-нейронов из обонятельной плакоды в передний мозг в процессе эмбрионального развития [1].

В последние годы благодаря развитию молекулярной биологии и внедрению технологий секвенирования следующего поколения (NGS) достигнут значительный прогресс в изучении ВГГ, и к настоящему моменту известно более 40 генов, ассоциированных с данными состояниями [2]. Наряду с X-сцепленным рецессивным и аутосомно-доминантным вариантами наследования в структуре ВГГ выделяют также аутосомно-рецессивные формы, распространенность которых нередко зависит от «эффекта основателя» и может значительно варьировать в различных этнических группах. В этой связи заслуживает особого внимания рецессивная форма ВГГ, обусловленная дефектами гена GNRHR, кодирующего рецептор ГнРГ. По данным литературы, доля данной формы ВГГ варьирует в широких пределах, колеблясь от 2% [3] до 32% [4].

В настоящем исследовании нами представлено описание 16 пациентов с доказанными дефектами рецептора ГнРГ и оценена частота патогенных вариантов в гене GNRHR в российской популяции.

## ОПИСАНИЕ СЛУЧАЕВ

В группу пациентов с гипогонадотропным гипогонадизмом включены 175 пациентов (мальчики (n=139), девочки (n=36)). Этническая принадлежность обследуемых — русские.

Молекулярно-генетический анализ проводился в лаборатории отделения наследственных эндокринопатий ФГБУ «НМИЦ эндокринологии» Минздрава России. Геномную ДНК выделяли из лейкоцитов периферический крови наборами PureLink® Genomic DNA Mini Kit (Thermo Scientific, Waltham, MA, USA). Применялся метод таргетного секвенирования следующего поколения (NGS). Использовалась авторская панель «Гипогонадотропный гипогонадизм» (технология Ion Ampliseq™ Custom DNA Panel, Thermo Scientific, Waltham, MA, USA), содержащая праймеры для мультиплексной ПЦР и секвенирования кодирующих последовательностей следующих 30 генов: CHD7, DNMT3L, DUSP6, FGF17, FGF8, FGFR1, FLRT3, GNRH1, GNRHR, HS6ST1, IL17RD, INSL3, ANOS1, KISS1, KISS1R, LHB, NSMF, POLR3B, PROKR2, RBM28, SEMA3A, SPRY4, TACR3, WDR11, GREAT, TAC3, PROK2, NR0B1, POLR3A, MKRN3. Подготовка библиотек проводилась в соответствии с рекомендациями производителей. Секвенирование осуществлялось на полупроводниковом секвенаторе PGM (Ion Torrent, Thermo Scientific, Waltham, MA, USA). Биоинформатическая обработка результатов секвенирования проводилась с помощью программных модулей Torrent Suite 4.2.1 (Ion Torrent, Waltham, MA, USA). Для аннотирования вариантов нуклеотидной последовательности использовался пакет программ ANNOVAR ver. 2018Apr16 [5]. После анализа полученных данных проводилось подтверждение полученных мутаций методом Сэнгера на секвенаторе Genetic Analyzer Model 3130 (Thermo Scientific, Waltham, MA, USA). Для определения цис- или транс-положения пар гетерозиготных мутаций использовались данные обследования родителей, графический анализ прочтений BAM-файлов, а также TA-клонирование продуктов ПЦР с последующим секвенированием клонов методом Сэнгера. Оценка патогенности вариантов нуклеотидной последовательности проводилась согласно международным и российским рекомендациям [6][7]. Нумерации кодирующей последовательности генов GNRHR, ANOS1 даны по референсам NM_000406 и NM_000216 (http://www.ncbi.nlm.nih.gov/genbank) соответственно. Для сравнения частот нуклеотидных вариантов использованы данные gnomAD (https://gnomad.broadinstitute.org/) [8] и базы данных RUEXAC лаборатории ДНК-диагностики ФГБНУ «МГНЦ им. академика Н.П. Бочкова».

По результатам молекулярно-генетического исследования у 16 пробандов (мальчики (n=11); девочки (n=5)) выявлены изменения в гене GNRHR (табл. 1). У 15 пациентов клиническая картина соответствовала нормосмическому гипогонадотропному гипогонадизму, у 1 (пациент №4) — синдрому Кальмана (аносмия установлена клинически на основании жалоб).

**Table:** Таблица 1. Клинико-гормональные и молекулярно-генетические данные пациентов

№	Пол	Фенотип	ЛГ, Ед/л 0’ (240’)	ФСГ, Ед/л 0’	Тестостерон, нмоль/л	Эстрадиол, пг/мл	Ген	н/к	а/к	Аллельность	rs	gnomAD	RUEXAC
1	м	нГГ	0,44 (0,53)	0,15	0,01		GNRHR	c.G416A	p.R139H	hom	rs104893842	0,0001630	0,0014958
								c.T2C	р.М1T	het	rs768079550	0,00001812	0,0003740
2	м	нГГ	0,34 (9,8)	1,72	0,69		GNRHR	c.G416A	p.R139H	het	rs104893842	0,0001630	0,0014958
								c.G785A	р.R262Q	het	rs104893837	0,001789	0,0014958
3	м	нГГ	0,1 (0,1)	0,1	<0,17		GNRHR	c.G416A	p.R139H	hom	rs104893842	0,0001630	0,0014958
								c.T2C	р.М1T	het	rs768079550	0,00001812	0,0003740
4	м	СК	0,08 (-)	0,23	-		GNRHR	c.G416A	p.R139H	hom	rs104893842	0,0001630	0,0014958
								c.T2C	р.М1?	hom	rs768079550	0,00001812	0,0003740
							ANOS1	c.467delA	p.E156Gfs5X	hem	-	-	-
5	ж	нГГ	0,1 (0,1)	0,1		34	GNRHR	c.G416A	p.R139H	hom	rs104893842	0,0001630	0,0014958
6	м	нГГ	0,1 (2,9)	0,35	0,49		GNRHR	c.G785A	p.R262Q	hom	rs104893837	0,001789	0,0014958
7	м	нГГ	0,15 (0,6)	0,5	0,5		GNRHR	c.G416A	p.R139H	hom	rs104893842	0,0001630	0,0014958
								c.T2C	р.М1T	het	rs768079550	0,00001812	0,0003740
8	ж	нГГ	0,1 (0,25)	0,16		48,8	GNRHR	c.G416A	p.R139H	hom	rs104893842	0,0001630	0,0014958
								c.T2C	р.М1T	hom	rs768079550	0,00001812	0,0003740
9	ж	нГГ	0,23 (4,8)	0,42		63,3	GNRHR	c.G416A	p.R139H	het	rs104893842	0,0001630	0,0014958
								c.G785A	р.R262Q	het	rs104893837	0,001789	0,0014958
10	м	нГГ	0,1 (0,2)	0,1	0,3		GNRHR	c.G416A	p.R139H	hom	rs104893842	0,0001630	0,0014958
11	м	нГГ	0,4 (7,2)	0,87	0,67		GNRHR	c.G416A	p.R139H	het	rs104893842	0,0001630	0,0014958
								c.T2C	р.М1T	het	rs768079550	0,00001812	0,0003740
								c.G785A	р.R262Q	het	rs104893837	0,001789	0,0014958
12	ж	нГГ	0,55 (0,55)	0,47		<9,0	GNRHR	c.G416A	p.R139H	hom	rs104893842	0,0001630	0,0014958
13	м	нГГ	0,86 (10,3)	1,28	1,26		GNRHR	c.G416A	p.R139H	het	rs104893842	0,0001630	0,0014958
								c.A317G	p.Q106R	het	rs104893836	0,002749	0,0007479
14	м	нГГ	0,12 (0,4)	0,67	0,39		GNRHR	c.G416A	p.R139H	hom	rs104893842	0,0001630	0,0014958
15	м	нГГ	0,2 (0,2)	0,22	1,07		GNRHR	c.G416A	p.R139H	hom	rs104893842	0,0001630	0,0014958
								c.T2C	р.М1T	het	rs768079550	0,00001812	0,0003740
16	ж	нГГ	0,1 (1,53)	0,69	-	5	GNRHR	c.G416A	p.R139H	het	rs104893842	0,0001630	0,0014958
								c.A317G	p.Q106R	het	rs104893836	0,002749	0,0007479
								c.T2C	р.М1T	het	rs768079550	0,00001812	0,0003740

15 из 16 пациентов обратились с жалобами на выраженную задержку полового развития в подростковом возрасте (Ме 16,25 года); у одного пациента (№4) диагноз заподозрен в 9,5 года на основании сочетания аносмии с микропенисом и левосторонним крипторхизмом. Физическое развитие всех пациентов соответствовало норме. По данным рентгенографии костей кисти медиана костного возраста соответствовала 14 годам. Нарушение обоняния отмечалось с раннего возраста у пациента 4. Других фенотипических черт, характерных для синдрома Кальмана (дефекты средней линии, тугоухость, бимануальные синкинезы), ни у кого из обследованных выявлено не было.

По результатам гормонального исследования базальные уровни гонадотропинов соответствовали допубертатным значениям: Ме лютеинизирующего гормона (ЛГ) 0,15 Ед/л [ 0,1; 0,34 ]; Ме фолликулостимулирующего гормона (ФСГ) 0,445 Ед/л [ 0,2; 0,74 ]; Ме тестостерона (для мальчиков) 0,585 нмоль/л [ 0,32; 0,79 ], Ме эстрадиола (для девочек) 39 пг/мл [ 9; 48,8 ]. Всем пациентам старше 14 лет проведена проба с аналогом Гн-РГ, максимальный выброс ЛГ варьировал от 0,1 до 10,3 Ед/л (Ме 0,58 Ед/л [ 0,2; 4,68 ]).

В нашей когорте выявлено 4 варианта изменений нуклеотидной последовательности (табл. 1). Наиболее часто (n=15) идентифицирована мутация p.R139H. Сочетание данного варианта с p.M1T выявлено у 8 пациентов (во всех случаях оба варианта в цис-положении), с p.R262Q — у 4, с p.Q106R — у 2. У всех 16 пациентов определялись биаллельные изменения в гене GNRHR. Все нуклеотидные замены в гене GNRHR описаны ранее при изолированном гипогонадотропном гипогонадизме [9].

У 1 пациента (№4) биаллельная мутация в гене GNRHR сочеталась с ранее не описанной патогенной гемизиготной мутацией в гене ANOS1 (табл. 1).

## ОБСУЖДЕНИЕ

ГнРГ — гипоталамический декапептид, синтезируемый в нейронах медиобазальной и преоптической областей гипоталамуса и аркуатного ядра, имеет пульсаторный характер секреции и через гипоталамо-гипофизарную портальную систему попадает в переднюю долю гипофиза. ГнРГ осуществляет свое периферическое действие, связываясь со специфическим рецептором (GNRHR) в передней доле гипофиза [10].

Ген GNRHR (OMIM #138850) картирован на длинном плече хромосомы 4 (4q13.2) в 1994 г. (Kaiser и соавт.) [11], состоит из трех экзонов и двух интронов и кодирует рецептор ГнРГ 1 типа (рГнРГ), состоящий из 328 аминокислот и имеющий молекулярную массу 37 731 Да. рГнРГ относится к семейству G-белок-ассоциированных рецепторов, содержит семь трансмембранных доменов и внеклеточный 35-аминокислотный N-концевой домен с двумя предполагаемыми сайтами гликозилирования. Интересно, что этот белок не имеет С-концевого цитоплазматического хвоста, что делает процесс его интернализации относительно медленным [12].

Экспрессия рецептора определяется преимущественно в гонадотрофах передней доли гипофиза, а также в лимфоцитах, клетках молочных желез, яичников и предстательной железы. После связывания со специфическим лигандом рецептор взаимодействует с G-белком, в результате чего происходят активация фосфолипазы С и мобилизация внутриклеточного кальция [13]. Активация рецептора приводит к выбросу гонадотропинов — ЛГ и ФСГ, которые оказывают дальнейшее периферическое действие.

Фенотип нокаутных по Gnrhr мышей имеет сходства с ВГГ у человека и проявляется половым инфантилизмом, низким уровнем половых стероидов и гонадотропинов; помимо этого, отмечаются нарушения минерализации костной ткани и закладки зубов [13].

С момента первого описания мутации с полной потерей функции гена GNRHR в 1997 г. [14] на настоящий момент выявлено более 50 мутаций [http://evs.gs.washington.edu, 15], приводящих в основном к аминокислотным заменам, более редко — к образованию стоп-кодона либо делециям или инсерциям со сдвигом рамки считывания. Выявленные дефекты затрагивают как внеклеточный, так и трансмембранный и внутриклеточный домены рецептора [16–18].

В зависимости от степени нарушения функции рецептора пациенты имеют широкий спектр фенотипических проявлений — от синдрома фертильных евнухов и парциального гипогонадотропного гипогонадизма до более выраженных форм резистентности к ГнРГ, характеризующихся наличием крипторхизма, микропениса, неопределяемым уровнем гонадотропинов и отсутствием пубертата [13][17]. В нашей когорте у всех пациентов клинически определялись выраженные проявления резистентности к ГнРГ, и лишь в одном случае отмечался низконормальный выброс ЛГ на стимуляцию аналогом ГнРГ (пациент № 13). У данного пациента была выявлена компаунд-гетерозиготность по 2 мутациям (p.Q106R/p.R139H), первая из которых характеризуется частично сохраненной функцией рецептора по данным функционального анализа in vitro [14].

рГнРГ не связан с процессом миграции ГнРГ-нейронов из обонятельной плакоды, поэтому при мутациях в гене GNRHR не ожидается нарушение обоняния. Между тем поводом для первоначального обследования одного из наших пациентов (№ 4) была именно аносмия, и последующее молекулярно-генетическое обследование, помимо биаллельного дефекта в гене GNRHR, выявило также патогенный гемизиготный вариант в гене ANOS1, мутации в котором являются наиболее частой причиной синдрома Кальмана. Это наблюдение подчеркивает важность использования мультигенных панелей для выяснения этиологии ВГГ.

Несмотря на то что в данный момент известно много других генов, ассоциированных с развитием ВГГ, мутации GNRHR остаются самой частой причиной развития этого состояния [19]. Однако данные по частоте встречаемости дефектов в гене GNRHR у пациентов с ВГГ значительно варьируют, что определяется критериями включения пациентов в исследование, методом молекулярно-генетического анализа и, не в последнюю очередь, популяционными особенностями. Так, Quaynor и соавт. [3] в рамках многоцентрового исследования в США провели таргетное высокопроизводительное секвенирование с анализом 261 гена-кандидата у 48 пациентов с ВГГ (у 22 — СК) и выявили мутацию GNRHR (p.R35C) у 1 пациента (2,1%). В работе Beranova и соавт. [20], которые прицельно анализировали ген GNRHR у 108 пациентов из США с различными формами ВГГ, мутации были найдены в 5 случаях, что для всей когорты соответствовало доле 4,6%, а в подгруппе 48 пациентов с ВГГ и нормосмией — 10,4%. Как и ожидается для аутосомно-рецессивного типа наследования, наиболее часто изменения в гене GNRHR выявляются при семейных формах ВГГ. В пользу этого свидетельствует, например, публикация Gurbuz F. и соавт. [4], в которой в турецкой популяции было проанализировано 22 семейных случая ВГГ без аносмии, и при анализе ассоциированных с рецессивными формами ВГГ генов (GNRHR, GNRH1, TACR3, TAC3, KISS1R и KISS1) мутации в гене GNRHR выявлены в 7 семьях (31,8%). Интересно, что среди этих 7 семей только в 2 определялись варианты (p.R139H и p.R262Q соответственно), которые встречались в изученной нами когорте больных. Показательно, что, по данным gnomAD [8], общая частота для варианта p.Q106R является самой высокой (табл. 1), что также согласуется с результатами анализа гена GNRHR при ВГГ среди европейцев [9][18][20]. Между тем в нашем исследовании p.Q106R был найден только у 2 пациентов (оба случая — в составе компаунд-гетерозиготной мутации), а чаще выявлялась мутация p.R139H (у 15 пациентов). При оценке популяционной частоты варианта p.R139H по данным RUEXAC она оказалась приблизительно в 9 раз выше, чем частота данного варианта в gnomAD (табл. 1). Следует также отметить, что в 8 из 15 случаев вариант p.R139H был сцеплен (находился на одном аллеле) с заменой р.М1T, что также, по-видимому, является особенностью российской популяции.

## ЗАКЛЮЧЕНИЕ

Таким образом, нами представлен опыт применения высокопроизводительного параллельного секвенирования для молекулярно-генетической диагностики ВГГ. В обследованной нами когорте мутации в гене GNRHR были наиболее частой причиной данного заболевания, составив 9,1%. Обращает на себя внимание преобладание патогенного варианта p.R139H и его частое сочетание в цис-положении с вариантом р.М1T, что отличает полученные нами данные для российских больных от частот вариантов в гене GNRHR при ВГГ, описанных в других популяциях.
